# Rationale and Description of Implementation of Regional Collaborative Service Model for Enhancing Psychosocial Wellbeing of Children and Families—Oulu Collective Impact Study

**DOI:** 10.3389/fpsyt.2022.784995

**Published:** 2022-03-07

**Authors:** Tuula Takalo, Sami Räsänen, Helinä Hakko, Aapo Juutinen, Mika Niemelä

**Affiliations:** ^1^Research Unit of Clinical Neuroscience, Psychiatry, Faculty of Medicine, University of Oulu, Oulu, Finland; ^2^Department of Psychiatry, Oulu University Hospital, Oulu, Finland; ^3^Faculty of Medicine, Center for Life Course Health Research, University of Oulu, Oulu, Finland

**Keywords:** children, adolescents, families, collective impact, collaboration, implementation, Let's Talk about Children Intervention, Let's Talk about Children Service Model

## Abstract

**Background:**

The psychosocial wellbeing of children and adolescents is an ongoing global concern. Despite positive outcomes of child- and family-focused programs, the fragmentation of services presents challenges. To enhance harmonization and diminish fragmentation of service policies, we implement a preventive collaborative service model for children and families. The rationale for our study is based on analyses of national and local data before implementing the service model in the pilot area.

**Methods:**

The need for a preventive service model for children and families was demonstrated using national and local data sources. First, a national school health survey was utilized to screen adolescents' perceptions of their depressive symptoms and support. Second, time trends in child and adolescent psychiatric and child protection service use were investigated. For these aims, epidemiological data of the study area (city of Oulu) were compared with data from the whole country (Finland). Third, local usage data of the preventive stand-alone Let's Talk About Children (LT) intervention before the service model implementation were evaluated. After these analyses, Let's Talk About Children Service Model (LT-SM) implementation in the pilot area is described in detail.

**Results:**

The background data showed that 17% of the adolescents in the study area had reported depressive symptoms, and almost half of them had not received professional help. Child and adolescent psychiatric outpatient visits had increased during the last decade, but the number of visits remains lower in the study area compared with the country as a whole. The number of adolescent psychiatric inpatient days had increased contrary to a decreasing national trend. The number of urgent child welfare placements was also higher compared with the whole country. The local LT intervention data revealed very low utilization rates. These results indicated a necessity to enhance preventive and collaborative support for children and their families. This article describes the implementation of the LT-SM.

**Discussion:**

We demonstrated excessive use of curative services in social and health care and insufficient usage of the stand-alone preventive intervention. The LT-SM is now piloted in one regional service area of the city of Oulu. Its effectiveness will be evaluated when enough data have been accumulated for statistical analyses.

## Introduction

Recently, there has been an increase in child protection needs and acts in Finland, some Nordic countries, and in Europe as a whole. Studies and reports have documented increased rates of psychological symptoms, diagnosed mental illness, and the use of psychiatric and social services in persons younger than 18 years (1–4). It is unclear whether the increased use of curative services indicates a higher incidence of illnesses; research suggests that they may also be indicators of weaknesses, such as inefficiency and fragmentation, in the current child and family social and healthcare services (3).

Separate policies and lack of collaboration between service providers are associated with inefficiency of the service system (5–7). This is understandable, as it may be difficult to take into account individually varying needs, diverse family backgrounds, and differences between communities when arranging the required services (8, 9). In response to these challenges, in Finland, government-funded programs for social and healthcare, daycare, and schools have been launched over the last decade, and national and international evidence-based programs and interventions have been implemented (10–18). However, these approaches have been narrowly oriented, restricted, and usually limited to one service area (19).

The most recent solution to the inefficiency of services has been to develop comprehensive service models covering all public providers as well as non-profit organizations. Various stand-alone interventions are used in these models, but they are integrated into a larger entity serving the common goal shared by the sectors. Utilizing these comprehensive models is expected to enhance psychosocial wellbeing and decrease the need for curative healthcare services or custodial arrangements in social services (20). An encouraging initiative is the Let's Talk About Children Service Model (LT-SM), which is a community-based infrastructure model developed for reinforcing child and family wellbeing and resilience. The main principle is to create a cross-sectoral infrastructure from prevention to treatment across different services and hierarchies (21). The LT intervention is an essential stand-alone method within the LT-SM, and there is encouraging research evidence indicating that it enhances psychosocial wellbeing and prevents the intergenerational impact of parental problems, such as parental mental health conditions (17, 18, 22–24) and somatic illnesses (25, 26).

The LT-SM shares principles with the collective impact (CI) framework in organizing services introduced by Kania and Kramer (27). The CI framework includes five conditions: a common agenda, shared measurement system, mutually reinforcing activities, continuous communication, and backbone support organization (21, 27). CI has been reported to be utilized with varying initiatives, such as food, health, and education programs (20, 28–30), whereas the LT-SM is focused on children's psychosocial wellbeing (21, 31). However, both models are intended to decrease the fragmentation of and improve the collaboration between services (21, 27, 32).

Reliable research-based evidence of the effectiveness of comprehensive models is lacking. In practice, examining or making conclusions about a model's efficacy at cross-sectoral service and service user level is not possible until after comprehensive implementation of the model (30, 32). Therefore, it is important to thoroughly describe the implementation process of the CI framework-based LT-SM.

The rationale of our study is based on analyzing epidemiological data on the perceived need and use of child and adolescent health and social services in the study area (city of Oulu). In addition, numerical data from the study area on the use of a single, preventive stand-alone LT intervention for children, adolescents, and families in different services before the implementation of the LT-SM in the pilot area of the city of Oulu were evaluated.

First, as indicators of need of services, we explored adolescents' perceptions of their depressive symptoms and experiences of receiving support for these problems in the study area (city of Oulu) utilizing the national School Health Promotion (SHP) survey data. Second, we analyzed 10-year time trends in the study area in the use of child and adolescent psychiatric and child protection services. In these two aims, the study area was compared with the whole country. Third, we examined the local usage data of the stand-alone LT intervention before the implementation of the LT-SM in the pilot area. After these analyses of national and local data, we describe in detail the implementation process of the LT-SM in the pilot area aimed to enhance the psychosocial wellbeing of children and families in Oulu. The effectiveness of that model will be evaluated in follow-up studies after implementation and systematic utilization of the LT-SM.

## Methods

The methods and results sections are structured as four sections. The first section consists of analyses of the national survey data of adolescents' perceptions of their depressive symptoms and experiences on receiving support for these problems in the study area (city of Oulu). The second section includes analyses of national epidemiological data, including the study area, to assess and compare time trends in the need and use of children and adolescent social and psychiatric services. The third section includes evaluation of the stand-alone LT intervention based on the numerical usage data from local registers of the city of Oulu at the time before the implementation of the LT-SM. The fourth section includes a detailed description of the implementation process of the LT-SM, which is piloted in one regional welfare service area (WSA) of Oulu.

### Perceived Symptoms of Depression Indicating Need for Support (Section 1)

Data regarding perceived symptoms of depression and experience of receiving support for them among 14–16-year-old adolescents (eighth and ninth graders from comprehensive schools) were based on the nationwide SHP study conducted in the years 2017 and 2019 (33). The national- and regional-level data were available from the SOTKANET databank produced by the National Institute for Health and Welfare. The SHP is administered nationwide every second year, with data gathered with an anonymous and voluntary classroom-administered questionnaire. The topics in the questionnaire include living conditions, schoolwork, health, health-related behavior, and school health services.

#### Perceived Depressive Symptoms

The Patient Health Questionnaire 2 (PHQ-2) (34) utilized in the SHP is a self-report assessment for screening depression, its severity, and patient treatment response used to examine the data on depressive symptoms. These data allowed us to estimate the number of the adolescents with depressive symptoms. The PHQ-2 assesses loss of interest in activities and low spirits, depression, and feelings of hopelessness over the last 2 weeks, scored from 0 to 2 (0 = no symptoms, 2 = loss of interest and mood involvement).

#### Experience on Receiving Support

Experience of receiving support for symptoms of depression was demonstrated by four indicators, which were based on two questions in the school health survey. The first question is: “Have you been worried about your mood during the past 12 months?” [answers: (1) No; (2) Yes, and I have told someone about it; (3) Yes, but I have not told anyone about it]. Question 2 asks: “Have you received support and help concerning your mood during the past 12 months?” [answers: (1) Yes, a lot; (2) Yes, some; (3) No, but I would have needed it; (4) I have not needed any help], and it consists of four subsections: (1) from school adults (teacher, school health nurse, physician, psychologist, social worker); (2) from services outside school (health center, mental health services, youth services, etc.); (3) from your own parents; (4) from friends and relatives. Indicators in **Figure 2A** for school adults, services outside school, own parents, or friends and relatives were calculated based on response alternative 2 for question 1 (Yes, had been worried about mood during the past 12 months and had told someone about it) and question 2 response alternatives 1–2 (Yes, received a lot or some support and help from school adults, services outside school, own parents, or friends and relatives). Furthermore, experiences of not having received help for depressive symptoms from either school adults/services outside school or from own parents/friends or relatives in the last 12 months despite a perceived need for help were illustrated by two indicators (**Figure 2B**). The proportions were calculated based on respondents who answered question 2 with response alternative 3 (did not receive support or help, although would have needed it). The indicators do not include those respondents who responded to question 2 that they had not needed any help.

### Epidemiological Data for Need and Use of Child and Adolescent Services (Section 2)

Data regarding child and adolescent psychiatric services and the use of child protection were gathered from the SOTKANET databank produced by the Finnish Institute for Health and Welfare (THL; Sotkanet.fi).

#### Use of Child and Adolescent Psychiatric Services

The psychiatric inpatient care indicator reflects the number of days young people spent in psychiatric hospital care (i.e., all psychiatric inpatient wards in the public sector) per 1,000 persons of the same age. The psychiatric outpatient care indicator provides the number of outpatient visits within child (aged 0–12 years) and adolescent psychiatry (aged 13–17 years) per 1,000 persons of the same age. No psychiatric inpatient care was provided in the private sector.

#### Use of Child Protection Services

The child protection indicator per 1,000 population reflects the percentage of children and adolescents aged 0–17 years who received child welfare placements or urgent (emergency) child welfare placements, both voluntarily and involuntarily. This indicator also includes those placed in care who turned 18 years during that year.

### Use of the Stand-Alone LT Intervention (Section 3)

The stand-alone LT intervention (35) as a single intervention method has been implemented and registered in the city of Oulu since 2015 and 2017, respectively, Agreement for Wellbeing 2013–2017 (36). The LT intervention was offered universally to all parents of our target population. The numerical data for utilization of the LT intervention were extracted from the local statistical register of the city of Oulu including the Primus, Effica, and LifeCare register systems (37). These electronic register tools are intended for monitoring and collecting work-related information for professionals in daycare, schools, and services. The statistics on the use of the LT intervention were calculated by dividing the number of users by the total number of children in the target population, including public daycare, school, maturity and child health clinics, and school health services.

### Implementation (Section 4)

#### Model Applied in Implementation

The LT-SM is a community-based action model aimed to enhance child and family wellbeing and resilience and prevent child and family problems. This model includes specific preventive interventions. The LT intervention has two steps: LT Discussion with families mapping out and supporting the protective factors of the children and LT Network with cross-sectoral collaboration including the families and their social network (17, 18, 21, 31, 35). The LT-SM has two shared goals: to support children's everyday life in all developmental contexts and environments (i.e., at home, daycare, school, and leisure environments) and to build the corresponding service structure (21, 31).

A specific service structure was established with the following parts: (1) cross-sectoral Multiagent Management Group (MMG) with all core leaders from all relevant sectors, (2) a feedback system to collect information on implementation and collaboration quality, and (3) One Contact Service (OCS) to ensure that all families in the area would receive services and support within 1–2 weeks (21).

#### Pilot Region of Implementation

The implementation process was piloted in one (out of 18) regional WSAs in the city of Oulu, and it covered all social and healthcare services for children, adults, and families. It comprised three public schools (~2,500 students), six daycare units (~1,050 children), and a local healthcare center, including a well-child clinic. The participating daycare and school units included the school's student welfare services comprising nurses, social workers, and psychologists. The implementation process in the pilot area involved a total of 450 professionals: 150 from daycare, 260 from schools, and 40 social and healthcare professionals.

### Statistical Methods

**Figure 1:**
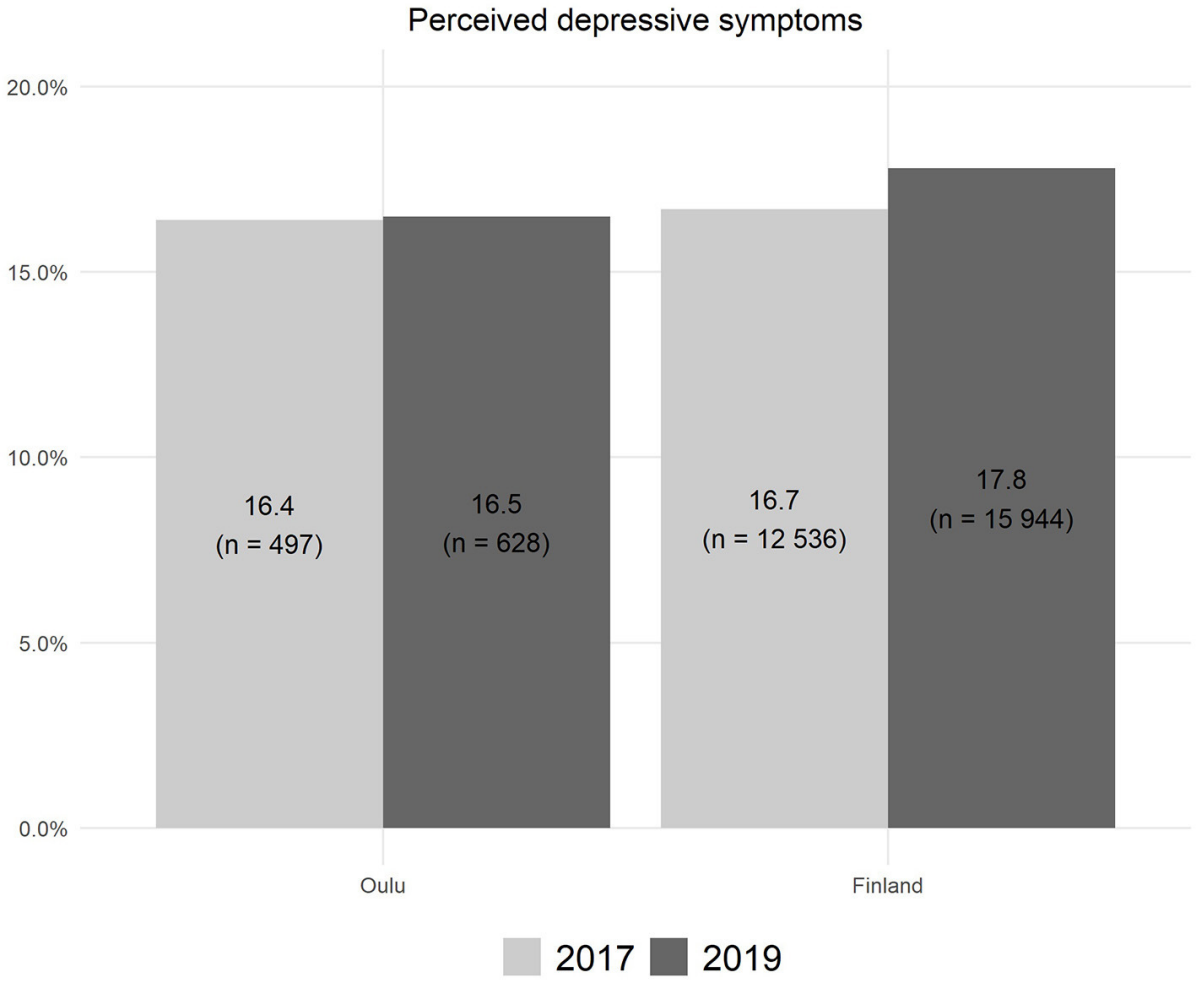
Perceived symptoms of depression lasting at least 2 weeks among 14–16-year-old pupils in Oulu and Finland in 2017 and 2019.

**Figure 2:**
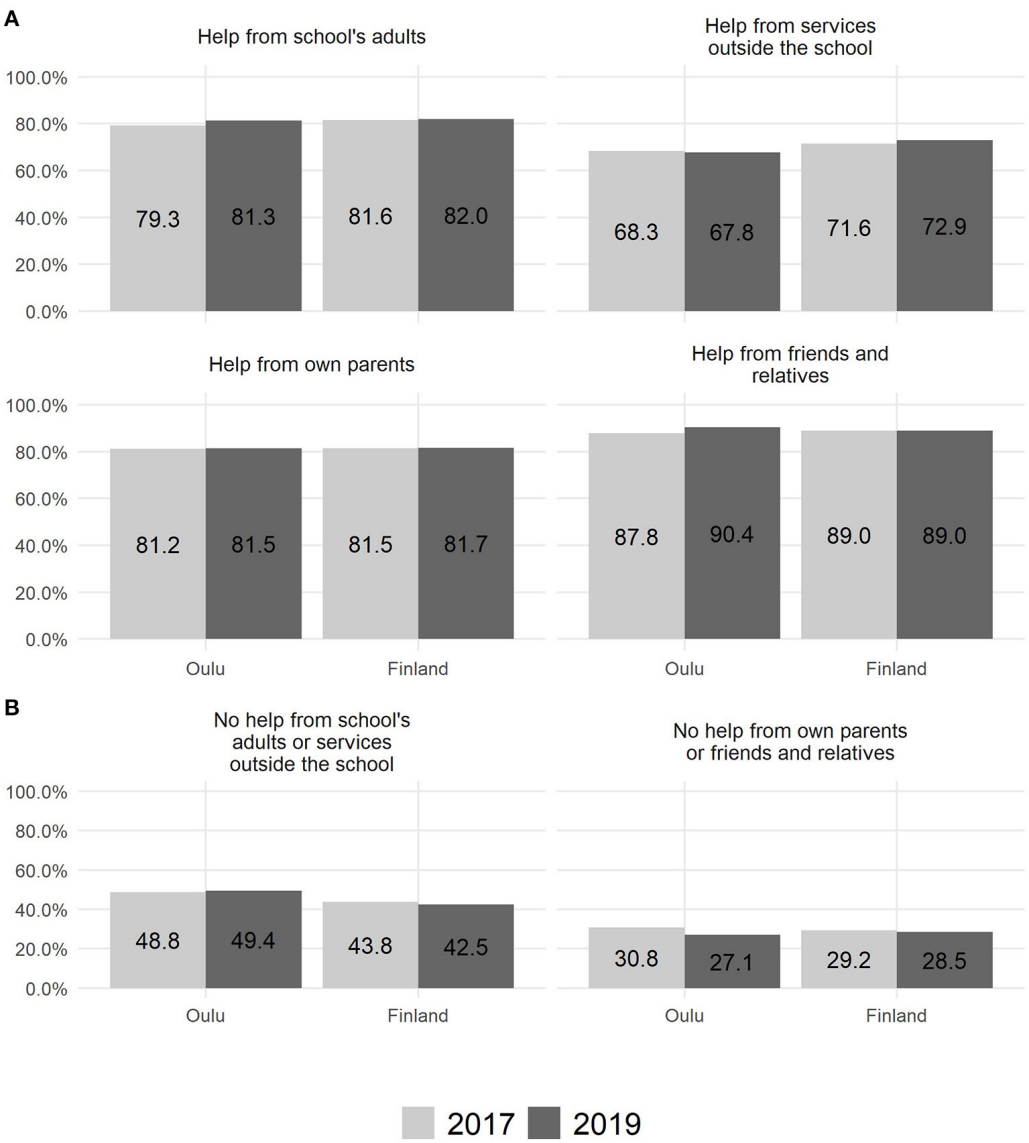
Experience of receiving **(A)** and not receiving **(B)** help for perceived symptoms of depression among 14–16-year-old adolescents (*n* = 628).

**Figure 3:**
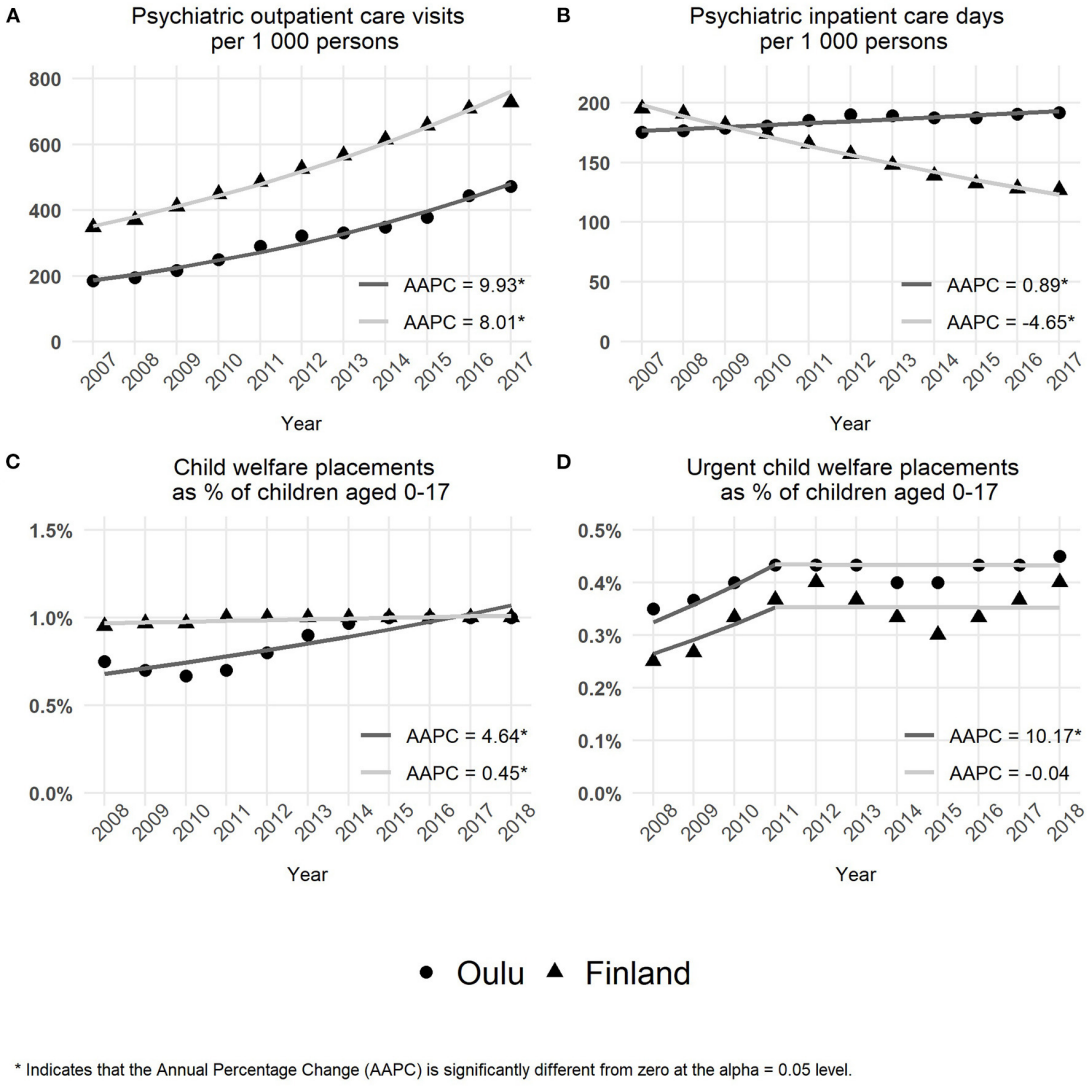
Use of child and adolescent psychiatric services and child protection services. **(A)** The number of outpatient care visits per 1,000 persons. **(B)** The number of inpatient care days per 1,000 persons. **(C)** The percentage rates of child welfare care placements. **(D)** The percentage rates of urgent child welfare care placement.

The Joinpoint Regression Program (version 4.8.0.1; Statistical Methodology and Applications Branch, Surveillance Research Program, National Cancer Institute, Bethesda, MD, USA) and the average annual percentage change (AAPC) was used to analyze time trends in the rates of psychiatric inpatient care days, outpatient visits, those placed in care, and those who received emergency placement, as well as to compare time trends between the city of Oulu and the whole country. Because of the relatively small number of observations causing high variability in the number of psychiatric inpatient care days and outpatient visits, a 3-year moving average (2-year moving average at the end of time periods) was used for the statistical modeling. The statistical significance of difference in AAPCs between the study area (city of Oulu) and the whole country (Finland) was calculated. In addition, the test of parallelism was used to determine whether the two regression mean functions between the study area and the whole country were parallel (same slope), allowing different intercepts. The remaining analyses and most of the Figures 1–3 were performed using RStudio (version 1.2.1335; RStudio, Boston, MA, USA).

## Results

### Perceived Symptoms of Depression and Received Support (Section 1)

In the study area (city of Oulu), a total of 3,028 (68% aged 14–16 years) adolescents in 2017 and 3,803 (76%) in 2019 participated in the nationwide school health promotion study. The corresponding figures for Finland as a whole were 75,068 (64%) in 2017 and 89,570 (75%) in 2019.

#### Perceived Depressive Symptoms

Approximately 16% (*n* = 497 and 628) of the adolescents reported symptoms of depression in the study area (city of Oulu) during 2017 and 2019, respectively (Figure 1). Accordingly, 16.7% (*n* = 12,536) and 17.8% (*n* = 15,944) of all Finnish adolescents reported depressive symptoms in 2017 and 2019, respectively. These proportions differed statistically significantly between Oulu and the whole country in the year 2019 (χ^2^ = 4.14, *p* = 0.042), but not in the year 2017 (χ^2^ = 0.17, *p* = 0.679).

#### Experience on Receiving Support

As illustrated in Figure 2, in the study area (city of Oulu), support was received from school adults (2017: 79.3%; 2019: 81.3%), services outside of school (2017: 68.3%; 2019: 67.8%), parents (2017: 81.2%; 2019: 81.5%), and friends and relatives (2017: 87.8%; 2019: 90.4%). The adolescents who reported a need for support for their depressive symptoms felt that they did not receive support from school adults or services outside school (2017: 48.8%; 2019: 49.4%) or from their parents, friends, and relatives (2017: 30.8%; 2019: 27.1%).

Nationally, adolescents received support from school adults (2017: 81.6%; 2019: 82.0%), services outside of school (2017: 71.6%; 2019: 72.9%), parents (2017: 81.5%; 2019: 81.7%), and friends and relatives (2017: 89.0%; 2019: 89.0%). There was also a substantial percentage of adolescents who perceived a lack of support from school adults or services outside school (2017: 43.8%; 2019: 42.5%) or from parents or friends and relatives (2017: 29.2%; 2019: 28.5%).

### Epidemiological Data on Need and Use of Child and Adolescent Services (Section 2)

The time trends over the years 2007–2017 in the need and use of child and adolescent services as well as the use of child protection services were based on the population-adjusted data obtained from the nationwide indicator databank. The results are presented in Figure 3.

#### Use of Child and Adolescent Psychiatric Services and Child Protection Services

As illustrated in Figure 3A, the rate of outpatient care visits showed a significant increase in both the study area (city of Oulu) (AAPC = 9.9; 95% confidence interval = 8.9–11.0; *p* < 0.001) and the whole country (AAPC = 8.0; 95% confidence interval = 7.5–8.5; *p* < 0.001). The AAPC of the study area was significantly higher compared with Finland (difference = −1.9; 95% confidence interval = −2.9 to −0.9; *p* < 0.001). The overall test for parallelism also revealed a statistically significant difference in time trends between the study area (city of Oulu) and the whole country (*p* = 0.028).

From 2007 to 2017, the rate of inpatient care days (Figure 3B) showed a significant increase in the study area (city of Oulu) (AAPC = 0.9; 95% confidence interval = 0.6–1.2; *p* < 0.001) compared with a decreasing trend in Finland (AAPC = −4.6; 95% confidence interval = −5.0 to −4.3; *p* < 0.001). Furthermore, there was a statistically significant difference in AAPCs between the study area and Finland (difference = −5.5; 95% confidence interval = −5.9 to −5.1; *p* < 0.001). Following the reverse time trends observed in the study area and Finland, the overall test for parallelism showed a statistically significant difference between the study area and the whole country (*p* = 0.002).

#### Use of Child Protection Services

As seen in Figure 3C, over the entire time period, the percentage rates of adolescents placed in care showed a significant increase in both the study area (city of Oulu) (AAPC = 4.6; 95% confidence interval = 2.9–6.4; *p* < 0.001) and the whole country (AAPC = 0.4; 95% confidence interval = 0.2–0.7; *p* = 0.005). The increase in AAPC of the study area was significantly higher compared with Finland (difference = −4.2; 95% confidence interval = −5.7 to −2.7; *p* < 0.001). In addition, the overall test for parallelism revealed a statistically significant difference in time trends between the study area and the whole country (*p* = 0.002).

As shown in Figure 3D, a significant change occurred in time trends in the year 2011. During the first 4 years from 2008 to 2011, the percentage rates of urgent placements showed a significant increase in both the study area (city of Oulu) and the whole country (AAPC = 10.2; 95% confidence interval = 1.0–20.1; *p* = = 0.031). After 2011 up to 2018, the percentage rates of urgent placements remained stable in both the study area and Finland as a whole (AAPC = −0.04; 95% confidence interval = −2.3 to 2.3; *p* = 0.969). Throughout the whole 10-year time period, from 2008 to 2018, the percentage rates of urgent placements showed a significant increase in the study area and Finland (AAPC = 2.9; 95% confidence interval = 0.06–5.9; *p* = 0.045).

### Use of Stand-Alone LT Intervention in Study Area (City of Oulu) (Section 3)

**Figure 4:**
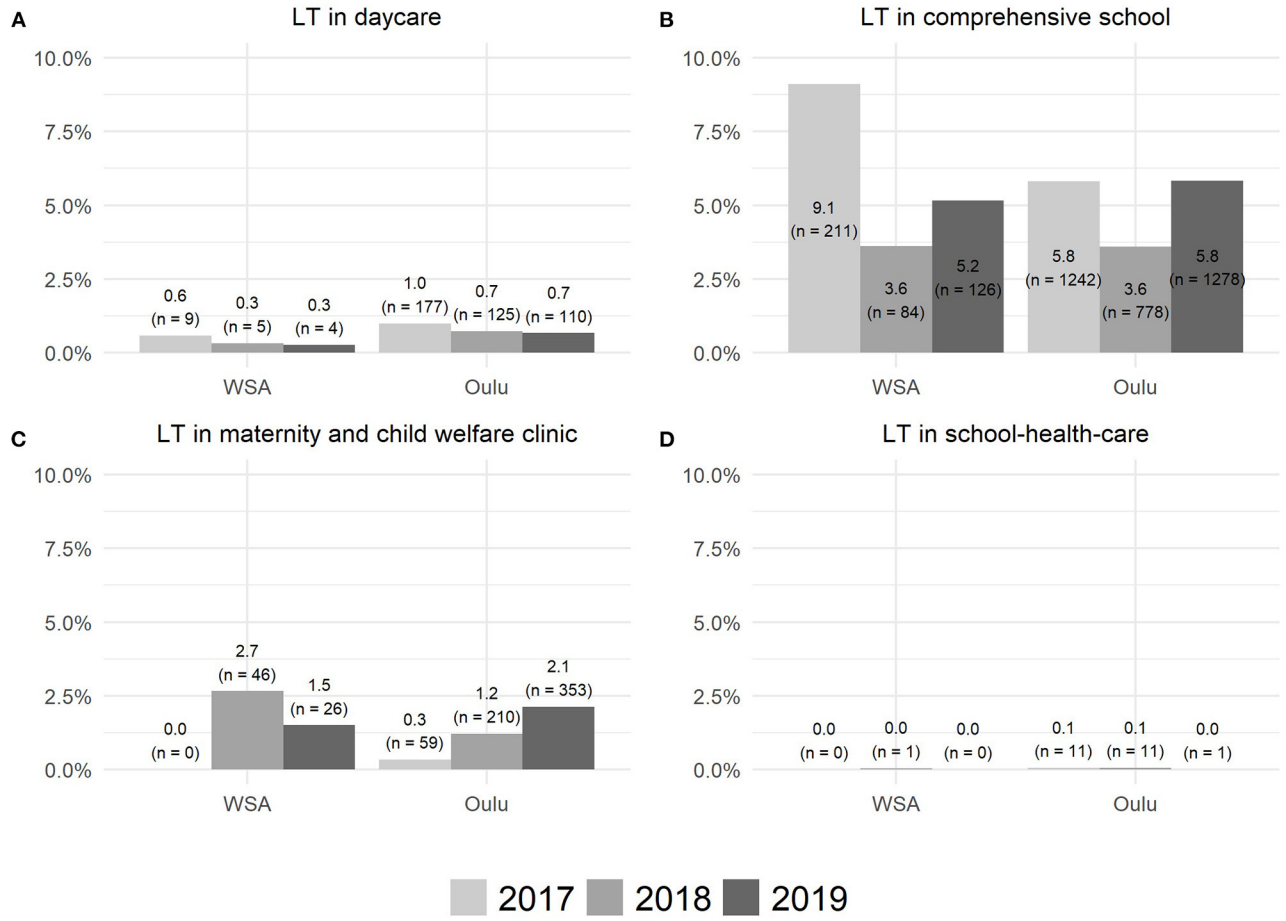
The use of the LT intervention in daycare **(A)**, comprehensive school **(B)**, maternity and child welfare clinic **(C)**, and school-health-care **(D)**.

As demonstrated in Figure 4, the use of the LT intervention varied between 0% (school healthcare) and 5.8 and 9.1% (comprehensive schools) in the study area (city of Oulu) and in the implementation pilot area including one WSA in Oulu, respectively.

### Description of CI Implementation Process (Section 4)

**Figure 5:**
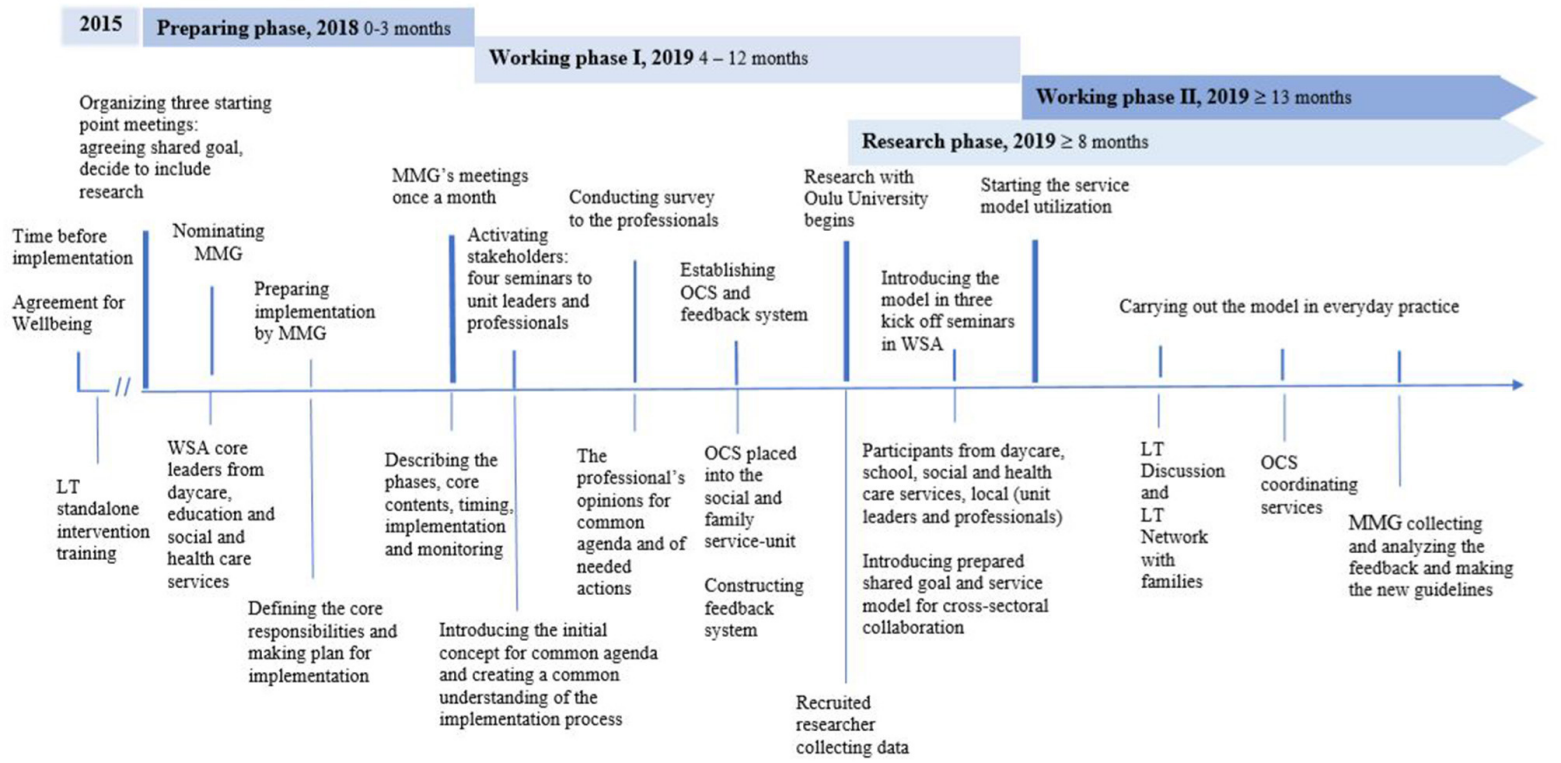
CI implementation process in 2018–2020. LT, Let's Talk About Children intervention; LT-SM, Let's Talk About Children Service Model; CI, collective impact framework; MMG, Multiagent Management Group; OCS, One Contact Service; WSA, the pilot area of Oulu, one regional welfare service area.

The CI implementation process was divided into three different phases. The starting point was the meeting where the common agenda was agreed. The phases from the starting point were the preparing phase (0–3 months), working phase I (4–12 months), and working phase II (13 months). The implementation research with the University of Oulu was prepared from the preparing phase of the process. It started 8 months after the starting point, during working phase I, once all necessary administrative permissions and funding for research had been obtained. The actions of the implementation are described in Figure 5.

#### Preparing Phase

The preparing phase began in October 2018. It included cross-sectoral meetings, nominating an MMG, creating a concrete action plan for implementation, and determining which research to include.

##### Organizing Starting Point Meetings

In October 2018, three starting point meetings were arranged to prepare for the implementation. The participants were the core local leaders of the pilot area (WSA) from daycare, education, social and healthcare services, and as backbone support, an external consultant with prior extensive experience in the LT-SM implementation process. At the conclusion of the meetings, commonly accepted shared goals were set: these comprised supporting children's everyday lives in all service sectors and in the following developmental environments: daycare, school, and leisure time. This goal was accepted by the division leaders of the city of Oulu and all local leaders, who committed to organize the required meetings, seminars, and trainings. In addition, scientific research was integrated into the implementation process from the start (Figure 5).

##### Nominating the MMG

In October 2018, the MMG consisting of core leaders from different sectors in the pilot area (WSA) was nominated, aiming to ensure that the units were committed to fulfilling the implementation tasks and providing consultation and support to the professionals and unit leaders. The MMG drew up a concrete step-by-step action plan on how to take into consideration the views of the unit leaders and professionals in all sectors. In addition, the MMG informed and organized training for professionals aimed to enhance and encourage communication between participants. The MMG was instructed to collect and analyze feedback from the units on a regular basis and perform all necessary actions based on the feedback.

#### Working Phase I

From January to June 2019, the MMG met six times (i.e., once per month). The MMG prepared and organized seminars to activate stakeholders from different sectors and explain which concrete tasks were intended to be carried out by particular professionals.

##### Activating Stakeholders

The first meeting for sharing information and mutual conversation was organized at the beginning of 2019. At least one leader or representative per unit and professionals from all services, daycare, schools, social and healthcare services, and student welfare services attended the meeting. During this meeting, the implementation plan prepared by MMG was introduced, and the proposed common agenda for the intended project was described.

Based on written feedback memos from the meetings, collaboration between daycare, education, and social and healthcare services was ranked according to importance by the participants. Eliminating delays in accessing child and family services was highlighted in particular.

During spring 2019, four information and open conversation meetings were arranged for the target group, which enabled a mutual understanding of what the implementation process required from the point of view of professionals, units, and organizations in different sectors. The background theories and core principles of the implementation were introduced. In particular, the professionals' opinions on how to develop the services were of interest.

##### Conducting the Survey

In spring 2019, the core stakeholders were surveyed with open questions administered to the professionals and units to determine their opinions of the necessary actions on conducting the implementation process. Altogether 61 responses were received from 9 unit meetings, with ~4–15 participants at each meeting. The participants were asked to describe how to enhance the availability of social and healthcare services and cross-sectoral collaboration between different service sectors in practice. The content was analyzed by bringing together the main themes that were most frequently brought up. The responses highlighted that (1) there is a need to build a shared unified service model because the current collaboration does not work appropriately and there is a need for coordination of the services; (2) cross-sectoral collaboration should be based on a common and united agenda to which all sectors will commit; (3) the commitments should be reliable and such that all participants can trust them; (4) there should be strict follow-up on how the guidelines of the model have been fulfilled; (5) the service model should enhance goal-oriented and responsible work; (6) the service model should allow families to be in key role in the collaboration; (7) there should be a simple method to invite professionals into the collaboration; and (8) the professionals emphasized the importance of knowing each other and of lively exchange of information between collaborative partners.

##### Establishing OCS

The MMG prepared the OCS model for the child and family services in the pilot area (WSA). The OCS was decided to be placed in the social and family services unit. Students' welfare services and social and healthcare services committed to participate in the LT Network meeting within a predetermined time. Urgent child protection actions (e.g., cases of family violence) continued as usual, according to the Child Protection Act.

##### Constructing Feedback System

The next task was to systematize the feedback system, which was initiated at the beginning of the implementation process. The feedback system served two purposes: (1) guiding and supporting professionals in different service sectors to fulfill their work according to the shared goal and (2) developing collaboration structures according to experiences accumulated from everyday work.

The feedback data (e.g., written feedback, experiences of LT Discussions, etc.) were collected by email from all units once per month before the MMG meeting. The MMG analyzed the feedback and the numerical usage data of the LT intervention and prepared the actions and new guidelines according to the feedback as necessary.

##### Introducing the LT-SM Model in Kickoff Seminars

After the preparation phase and working phase I, the LT-SM was introduced to all professionals in all service sectors: daycare, schools, social and healthcare services, and student welfare services. The concrete utilization of the model began on September 2, 2019.

The concrete service model was formed as follows:

When the professionals close to the child noticed stressful changes in a child's life (e.g., behavioral problems, parental illness, etc.), they were instructed to do the following:

1) Contact the child and parents: get a picture of the child's current situation.

2) Meet with children and parents, use LT intervention if possible, and include everyday adults in the child's life.

3) Use OCS, if necessary, for social and healthcare professionals: utilize required services.

4) Arrange an LT Network meeting: make concrete action plans for helping the child and family in collaboration with separate service sectors.

5) Arrange LT Network meetings with as many follow-ups as required: ensure reliable collaboration and services for the family.

In August 2019, the MMG organized three kick-off seminars to introduce the information to professionals in all sectors. The professionals from the OCS group participated in the seminars as well to familiarize them with future collaboration. The MMG introduced the prepared service model based on professional feedback and opinions. After the seminars, each unit was informed about the start of concrete CI service model utilization by sending out handouts with a detailed description of the main principles and agreed actions during the implementation process.

#### Research Phase

The research was included in the implementation process during working phase I. As a result of the negotiations with the University of Oulu, one researcher began to gather documentation data of the implementation process and baseline statistics before the implementation.

#### Working Phase II

Working phase II was initiated in September 2019. In working phase II, the aim was to conduct everyday practice and management as discussed. Thus, LT Discussions were meant to be conducted with families when there were changes in the family situation or problems with the child. In addition, OCS was to coordinate the required services to support the child and the close adults to manage these challenges. MMG collected and analyzed monthly feedback to develop the collaboration further.

## Discussion

Psychosocial wellbeing of children and adolescents is an ongoing societal concern (38, 39). Despite various local and national efforts and programs to promote the psychosocial wellbeing of young people, several child and adolescent health and social care indicators demonstrate concerning trends. Although child- and family-focused programs have generally reported positive outcomes (10–12), they have been unable to respond to the challenges caused by the fragmentation of services and separate service models. These concerns are acknowledged as a major contributor to the problems in the service sector.

In the present study, we describe in detail the implementation of the LT-SM in the pilot area, one regional WSA of the city of Oulu. This model is assumed to unify policies and solve major problems caused by the fragmented services. The follow-up data and forthcoming analyses of the model will produce research-based evidence on the efficacy of such a model. To demonstrate the rationale for the implementation of this model, we first evaluated various population-based child and adolescent health and social care indicators and the results of a nationwide school-health survey in the study area (city of Oulu) in comparison with those of the average of the whole country. We also explored the numerical usage data of the stand-alone LT intervention in the study area (city of Oulu) in the period before the implementation of the LT-SM.

We found that the number of child and adolescent psychiatric inpatient days has increased in the study area (city of Oulu), whereas in the whole country, the respective rates have decreased. Furthermore, psychiatric outpatient visits have increased in this area, but the level has remained lower when compared with that in Finland as a whole. These findings are consistent with the previous reports of increased mental health service use among children and adolescents (40–42). A high level of use in inpatient psychiatric services and a nationally low level in outpatient service use in the study area are a concerning finding and advocate for more effective preventive and outpatient-oriented service approaches. In addition to healthcare services, urgent child welfare placements by social services were at a notably higher level in the study area compared with the average of the whole country. This is alarming because the rates of child welfare placements in Finland are already high in international comparisons, and they are also regarded as too high by national professional and scientific communities (4, 38, 39). This alarming finding from the study area (city of Oulu) may be due to the nationally low rates of use of children and adolescent outpatient mental health services in the city of Oulu. Primarily, this may suggest not only insufficient levels of services in this area, but also unwillingness to seek help.

We found that nationally, approximately one-fifth of the adolescents who responded to the nationwide SHP survey reported that they suffered from depressive symptoms. A large majority of respondents reported that they received help from their close relatives and friends. With regard to professionals, adolescents reported most commonly that they received support from school personnel, such as teachers and school welfare professionals, but less commonly from social and healthcare services. On the other hand, almost half of the respondents who reported a need for help with their depressive symptoms had not received professional help. This lack of support was more common among adolescents from the study area (city of Oulu) compared with the national average. This indicates a lack of necessary services. The perceived need for support among adolescents is consistent with the increased trends observed in the use of adolescent social and healthcare services (40–42).

A worrisome finding was that before the implementation of the comprehensive LT-SM, the use of the stand-alone LT intervention in the study area (city of Oulu) as a single preventive method decreased to the minimum level soon after its initiation. This finding confirmed the previously known challenge of the implementation of stand-alone interventions, that is, that single interventions rarely remain part of everyday practice despite proper training and other implementation efforts (43).

Furthermore, our results on the epidemiological data are consistent with the previously internationally recognized need for preventive psychosocial and outpatient mental health services for children and adolescents (44, 45). The city of Oulu has attempted to respond to these challenges of preventive work with local actions (46) and by participating in national child and family development programs (10–12). The current study clearly demonstrated that despite these actions, the use of institutionalized services among adolescents has remained at high level. At the national level, the need for cross-sectoral collaboration between professionals has been a subject of ongoing debate. Even in Finnish legislation, there are regulations calling for collaboration between service sectors in areas such as education, student welfare, and social and healthcare (Basic Education Act 628/1998; Student Welfare Act 1287/2013; Social Welfare Act 1301/2014; Healthcare Act 1326/2010). However, there is no clear roadmap for professionals from different sectors on how to fulfill these requirements.

According to Kania and Kramer (27), CI framework initiatives have successfully established collaboration between services surrounding various initiatives such as HIV prevention and food and obesity programs (20). In Finland, promising results have been obtained from psychosocial children and family-focused preventive work when applying the LT-SM based on the CI framework (21).

In the implementation process of the LT-SM described in this article, the common agenda (i.e., supporting the everyday life of children and families) on collaboration with social and health care services and developmental environments was determined at the beginning of the preparation phase. The implementation process in the pilot area followed the principles of CI-related literature, which highlights the significance of a common agenda as a first condition for successful implementation. It is usually determined by core actors around the same topic who have connections with relevant stakeholders (47–49).

In the pilot area of Oulu, as part of the implementation process, the MMG collected monthly feedback from all collaborative units. Feedback-related communication in the MMG revealed the need for the required actions, including various common agenda-related discussions with professionals on how they can apply the common goal in their everyday work in different sectors. This was carried out in accordance with previous research literature where feedback-based measurement and communication were regarded as essential to lead the CI process and build collaboration (29, 50, 51).

In the current implementation process, the role of the OCS (20) was emphasized in terms of reinforcing activities (27). OCS invited the appropriate participants to the LT Network meetings based on contacts (e.g., from school and daycare). Consequently, the professionals in the OCS became aware of the needs and services required for the families. This information, together with general feedback collected from the units and service use statistics, helped the MMG focus on necessary activities. The OCS helped to integrate the actions of different services by inviting professionals to collaborate during network meetings. In summary, the OCS provided a possibility to increase integration and prevention within the services.

The role of backbone organization was essential in the implementation process in the pilot area of Oulu, which is consistent with previous recommendations in studies highlighting the need of backbone in collaborative organizations (20). Overall, backbone organizations are related to project funding (52), non-governmental and intermediary organizations to collaboration with various actors (53), and local administration to relevant stakeholders (54). In the pilot area, the MMG was the backbone of the whole organization because its members knew the whole implementation process, were able to facilitate the required actions, and monitor that all CI conditions were fulfilled.

The literature of the CI framework recognizes four steps in the change of collaboration into practice: (1) fulfilling the five CI conditions, (2) early changes and their connections with CI conditions, (3) systems changes in the core organizations and their connections with early changes and CI conditions, and (4) population changes and their connections with systems changes (55). The implementation process described in the current article is the first step toward the change of collaboration between the service sectors. Thus, profound population-level changes, such as a decrease in the rates of child welfare placements and adolescent psychiatric hospitalizations in the city of Oulu, will take time (30, 55).

### Strengths and Limitations

To our knowledge, this is the first detailed description of the CI implementation process aimed at child and family service integration in Nordic WSAs. The nationwide register data on the use of child and adolescent services as well as survey data on depressive symptoms and support perceived by adolescents were obtained from the SOTKANET databank, which has been acknowledged to be reliable for research purposes (56). This epidemiological data were related to the period prior to the implementation of the LT-SM in one WSA of the city of Oulu. The numerical data on the use of the stand-alone LT intervention in the pilot area were very limited and do not allow more detailed analyses, such as how many parents started using the model but did not finish it. The number of cases for Figure 2 was not available for our study. However, we believe that Figure 2 serves well to illustrate that the majority of the adolescents had received support and help from someone in their everyday life, but there is also a notable number of adolescents reporting need for help with their mental health who have not received it.

The present study clearly demonstrated a high rate of use of curative services in social and health care and insufficient usage of the stand-alone preventive intervention. The process described in this article indicates that a comprehensive CI-based service model can be implemented in the municipal service system, including all existing sectors. In this way, separate services are likely able to act as an integrated service entity. The effectiveness of the service model will be evaluated in the future when enough follow-up data have been accumulated for justifying reliable statistical analyses.

## Data Availability Statement

The raw data supporting the conclusions of this article will be made available by the authors, without undue reservation.

## Author Contributions

TT and MN were responsible for the implementation process. TT and MN wrote the first draft of the article. SR provided consultation during the implementation process, including research into it, and made significant contributions to the manuscript. HH and AJ were responsible for statistical analyses, including reporting and interpretation of the results based on these analyses. All authors participated in critical drafting of the manuscript and approved the final version of the manuscript and agreed to be accountable for all aspects of the work to ensure that questions related to the accuracy or integrity of any part of the work are appropriately investigated and resolved.

## Funding

The scholarship of TT (2020–2021) and professorship of MN (2020–2023) have been funded by ITLA Children's Foundation.

## Conflict of Interest

The authors declare that the research was conducted in the absence of any commercial or financial relationships that could be construed as a potential conflict of interest.

## Publisher's Note

All claims expressed in this article are solely those of the authors and do not necessarily represent those of their affiliated organizations, or those of the publisher, the editors and the reviewers. Any product that may be evaluated in this article, or claim that may be made by its manufacturer, is not guaranteed or endorsed by the publisher.

## Further Reading

Nordic Social Statistical Committee. Social Protection in the Nordic Countries 2013/2014. Scope, Expenditure and Financing. Nordic Social Statistical Committee (2015) (NOSOSCO) 60:2015. Copenhagen, Denmark. [Accessed 5.10.2020]. Available at: https://norden.diva-portal.org/smash/get/diva2:1059435/FULLTEXT02.pdf.

Social welfare act 1301/2014. [Accessed 30.11.2020]. Available at: https://finlex.fi/en/laki/kaannokset/1982/en19820710. Finland: The Ministry of Social Affairs and Health.

Basic education Act 628/1998. [Accessed 30.11.2020]. Available at: https://finlex.fi/en/laki/kaannokset/1998/en19980628?search%5Btype%5D=pika&search%5Bkieli%5D%5B0%5D=en&search%5Bpika%5D=628%2F1998. Finland: The Ministry of Education and Culture.

Health care act 1326. [Accessed 30.11.2020]. Available at: https://www.finlex.fi/en/laki/kaannokset/2010/en20101326?search%5Btype%5D=pika&search%5Bkieli%5D%5B0%5D=en&search%5Bpika%5D=1326%20%2F%202010. Finland: The Ministry ofSocial Affairs and Health (2010).

Student welfare act 1287. [Accessed 30.11.2020]. Available at: https://www.finlex.fi/en/laki/kaannokset/2013/en20131287?search%5Btype%5D=pika&search%5Bkieli%5D%5B0%5D=en&search%5Bpika%5D=student%20Welfare%20. Finland: The Ministry of Education and Culture (2013).

Social and healthcare services in Finland: Expert Evaluation. Autumn 2018. (ed.) Rissanen, P. 5/2019. [Accessed 30.10.2020]. Available online at: https://urn.fi/URN:ISBN:978-952-343-360-1

sotkanet.fi. Statistical information on welfare and health in Finland. [Accessed 5.10.2020]. Available at: https://sotkanet.fi/sotkanet/en/haku?g=354. National Institute for Health and Welfare(2020).

The City of Oulu. The Department of Educational and Cultural Services and the Department of Well-Being Services. The City of Oulu. Non-Published Material, 2017–2019 (2020). The statistics of the Let's Talk about Children Intervention years.
